# Deep Learning for Whole-Slide Tissue Histopathology Classification: A Comparative Study in the Identification of Dysplastic and Non-Dysplastic Barrett’s Esophagus

**DOI:** 10.3390/jpm10040141

**Published:** 2020-09-23

**Authors:** Rasoul Sali, Nazanin Moradinasab, Shan Guleria, Lubaina Ehsan, Philip Fernandes, Tilak U. Shah, Sana Syed, Donald E. Brown

**Affiliations:** 1 Department of Systems and Information Engineering, University of Virginia, Charlottesville, VA 22904, USA; rs8wa@virginia.edu (R.S.); nm4wu@virginia.edu (N.M.); 2 Department of Internal Medicine, Rush University Medical Center, Chicago, IL 60612, USA; shan.guleria@gmail.com; 3 School of Medicine, University of Virginia, Charlottesville, VA 22903, USA; le7jg@virginia.edu (L.E.); philipfern@gmail.com (P.F.); 4 Hunter Holmes McGuire Veterans Affairs Medical Center, Richmond, VA 23249, USA; tilak.shah@gmail.com; 5 Division of Gastroenterology, Hepatology and Nutrition, Virginia Commonwealth University, Richmond, VA 23219, USA; 6 School of Data Science, University of Virginia, Charlottesville, VA 22904, USA

**Keywords:** deep learning, whole-slide tissue histopathology, feature extraction approaches, Barrett’s esophagus

## Abstract

The gold standard of histopathology for the diagnosis of Barrett’s esophagus (BE) is hindered by inter-observer variability among gastrointestinal pathologists. Deep learning-based approaches have shown promising results in the analysis of whole-slide tissue histopathology images (WSIs). We performed a comparative study to elucidate the characteristics and behaviors of different deep learning-based feature representation approaches for the WSI-based diagnosis of diseased esophageal architectures, namely, dysplastic and non-dysplastic BE. The results showed that if appropriate settings are chosen, the unsupervised feature representation approach is capable of extracting more relevant image features from WSIs to classify and locate the precursors of esophageal cancer compared to weakly supervised and fully supervised approaches.

## 1. Background

Barrett’s esophagus (BE) is a precancerous condition that results from damage to the lining of the squamous esophageal mucosa. BE diagnosis is based on the endoscopic and histologic findings of the columnar epithelium lining the distal esophagus [1]. In order to increase sensitivity for dysplasia, guidelines recommend the Seattle protocol, which involves taking four-quadrant random biopsies at 1–2 cm intervals [2]. However, this protocol does not permit real-time diagnosis or therapy and is labor-intensive, leading to low adherence [3,4]. Additionally, numerous studies have documented poor inter-observer agreement among pathologists when diagnosing both low-grade [5,6,7] and high-grade dysplasia [8], suggesting significant room still exists for improvement in even the gold standard of histopathologic diagnosis. As dysplastic and non-dysplastic BE can progress to esophageal cancer, the need for an accurate and efficient diagnostic tool is evident. At the time of diagnosis, this knowledge could radically improve our clinical care by altering disease management and preventing disease complications. Therefore, there is a major need to develop innovative computational methods to translate heterogeneous histopathological images into accurate and precise diagnostics. The development of such a methodology in high-dimensional clinical research will support precision medicine, with improved diagnostics, predictions, treatments, and patient clinical outcomes. The success of these approaches relies on the appropriateness of extracted morphological features for characterizing the images.

There are some problems associated with conventional feature engineering approaches: First, the combinatorial nature of the feature extraction process makes it expensive to hand-craft features. In addition, the development of these features commonly relies on task/domain-specific expertise, preventing them from adapting to new tasks or domains. Furthermore, human bias is an inseparable part of hand-crafted features. In recent years, deep learning approaches have revolutionized the process of feature extraction tasks [9]. However, dealing with whole-slide tissue histopathology images (WSIs) offers new challenges, which demands more effective representation-learning approaches. Some challenges associated with these images include image size (typically 100,000 × 100,000 RGB pixels), high complexity, high morphological variance, and uncertainty associated with the pathology level. Automatic analysis of WSIs with typical deep learning approaches is impractical or impossible due to the above hurdles. Most recent researches has considered feature acquisition from high-resolution tissue tiles sampled from WSIs as a potential solution to these hurdles. In this approach, the final label of a given WSI is predicted based on image features extracted from sampled tissue tiles [10,11,12,13,14]. There is a rich body of literature investigating feature representation in the form of three primary approaches: fully supervised (FS) [15,16,17], weakly supervised [18,19,20,21,22,23,24], and unsupervised feature learning [25,26,27,28,29,30].

Of these, the fully supervised feature learning approach requires a large amount of accurately annotated data, which can be a labor-intensive, time-consuming, and error-prone process. These challenges are abundantly clear in the classification and segmentation of histopathology images as accurate and complete annotations can be difficult even for expert pathologists. On the opposite end of the annotation spectrum, unsupervised feature representation approaches aim to learn a discriminative representation of WSIs from annotation-free histopathology images according to the application domain. These methods extract the salient features from WSIs without requiring any image-level diagnosis as an image label or region of interest annotated by experts. Finally, weakly supervised methods have the advantages of both the fully supervised and unsupervised approaches for feature learning [31]. These approaches are not as highly dependent on annotated images as fully supervised approaches, nor are they as prior free as unsupervised approaches. Weakly supervised approaches exploit coarsely grained annotated WSIs to simultaneously classify histology images and yield pixel-wise localization scores, thereby identifying the corresponding regions of interest. Weakly supervised approaches in this way address the challenges related to the scarcity of densely annotated images.

This paper provides a comparative study that sheds light on the characteristics and behavior of different representation learning approaches through the sliding window approach, and their aggregation, by using a histogram-based method [32] for whole-slide inference, and identifying dysplastic and non-dysplastic BE on high-resolution histopathological images.

## 2. Materials and Methods

### 2.1. Data Collection

This study utilized previously published preliminary data to apply deep learning techniques for detecting BE and dysplasia in Hematoxylin and Eosin (H&E) stained biopsies. All patients in the study conducted by Shah et al. [6] (years 2014–2016) underwent targeted biopsy or mucosal resection, and Seattle protocol biopsies. In order to increase the sample size, a retrospective chart review was conducted to identify and retrieve biopsy slides of patients who had undergone upper endoscopies for BE surveillance (years 2016–2019). These patients all underwent high-definition white-light endoscopy (HD-WLE), narrow-band imaging (NBI), and acetic acid chromoendoscopy followed by targeted biopsies/mucosal resection, and Seattle protocol biopsies. All biopsy specimens were fixed in formalin. Samples were embedded to exhibit the full mucosal thickness. The paraffin blocks were sectioned at three microns to create biopsy slides that were stained with hematoxylin and eosin. All suspected diagnoses of dysplasia or malignancy required a consensus of two or more pathologists. For patients included in Shah et al.’s study [6], a blinded expert pathologist also reviewed all biopsy specimens. Blinded and unblinded pathology results were prospectively recorded.

This study was approved by the Hunter Holmes McGuire Veterans Affairs Medical Center Institutional Review Board and the University of Virginia Institutional Review Board for Health Science Research (IRB-HSR #21328).

### 2.2. Esophageal Biopsy Datasets

**Figure 1:**
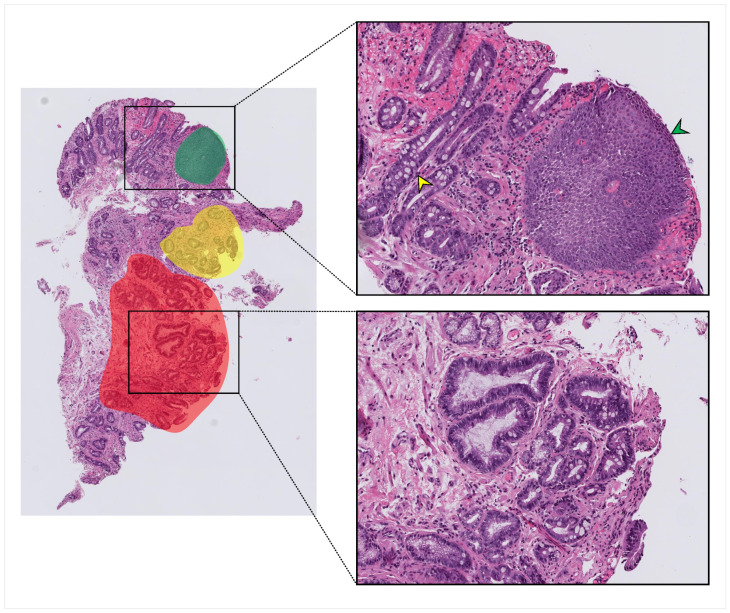
An example of the annotation process on a typical whole-slide image (WSI). Red, green, and yellow highlighted areas indicate areas that were annotated and from which labeled patches were taken. Squamous tissue (green arrowhead), non-dysplastic Barrett’s with Goblet cells (yellow arrowhead), and dysplastic tissue with crowding and hyperchromasia (lower zoomed section) were all present within the same whole-slide image.

Tissue images were digitized at 40× magnification via scanning of biopsy slides using a Hamamatsu NanoZoomer S360 Digital slide scanner C13220 [33]. A total of 387 whole-slide images from 130 unique patients were collected. WSIs increased to 650 after pre-processing and cropping; 115 whole-slide images from 10 patients were selected to train deep models to extract patch-level image features in all three feature learning approaches, and the rest of the dataset was used for model evaluation. To train deep models in fully supervised approaches, these WSIs were manually pixel-wise annotated to highlight each class’ examples within each whole-slide image (see Figure 1).

### 2.3. Deep Learning-Based Feature Representation

As a result of using the histogram-based method, each image is represented as a histogram; the mapping mechanism and histogram structure depend on the feature extraction approach (i.e., fully supervised, weakly supervised, or unsupervised). To extract histological features from tissue tiles, each WSI $X_{i},i=1,...,N$ is considered as a set of tissue tiles $X_{i}=\left\{x_{1},...,x_{n_{i}}\right\}$. This method uses a function M to map each image tile *x* belonging to WSI $X_{i}$ to a concept $c_{k},k=1,...,K$. In practice, instead of $x\in R^{D}$, its representation learned by neural network $f_{\psi }\left(.\right)$ with parameter ψ is considered, $f_{\psi }\left(x\right)\in R^{E}$ in which E<<D. All concepts can be organized in as a vocabulary $V=\{c_{1},...,c_{K}\}$. Consequently, WSI $X_{i}$ is mapped to histogram $H_{i}=\left(h_{1},...,h_{K}\right)$ as follows [32]:

(1) $$H_{i}=\frac{1}{|X_{i}|}\sum_{x\in X_{i}}M\left(f_{\psi }\left(x\right),V\right)\;\;\;\;i=1,...,N.$$

The *k*-th bin in $H_{i}$ is calculated as follows:

(2) $$h_{k}=\frac{1}{|X_{i}|}\sum_{x\in X_{i}}p\left(c_{k}|f_{\psi }\left(x\right)\right)\;\;\;\;k=1,...,K.$$

Here, $p(c_{k}|f_{\psi }\left(x\right))$ is the likelihood that embedding vector of tissue tile *x* belongs to $c_{k}$. In other words, the image-level histogram is the normalized frequency of each concept $c_{k}$ in the corresponding image.

#### 2.3.1. Fully Supervised Feature Learning

**Figure 2:**
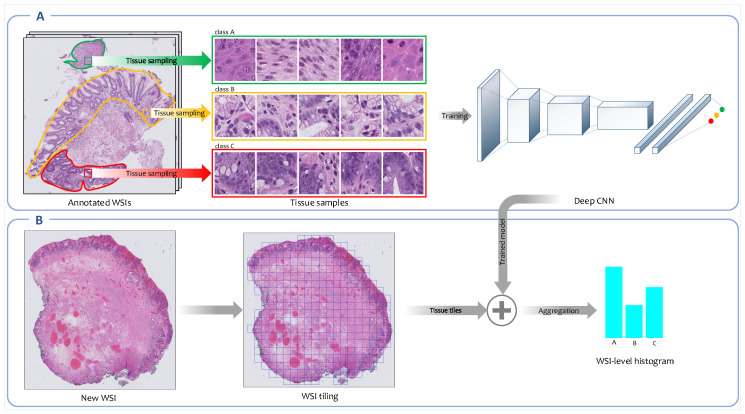
Overview of the fully supervised feature extraction framework. (**A**) A convolutional neural network (CNN) is trained on high-resolution tissue tiles sampled from annotated regions of WSIs in the training set. (**B**) Next, the trained model is employed to output the class’ probability distributions for each high-resolution tissue tile generated from new WSIs. The patch-level probabilities corresponding to all patches derived from a WSI are aggregated into WSI-level probabilities histogram.

In this approach, a convolutional neural network (CNN) is trained on high-resolution tissue tiles sampled from annotated regions of WSIs in the training set. Here, vocabulary $V=\{c_{1},...,c_{K}\}$ is the set of *K* classes and mapping function M is the fully connected classifier network of the CNN which outputs class probabilities $M\left(f_{\psi }\left(x\right),V\right)=\left(p\left(c_{1}|f_{\psi }\left(x\right)\right),...,p\left(c_{k}|f_{\psi }\left(x\right)\right),...,p\left(c_{K}|f_{\psi }\left(x\right)\right)\right)$ for each input tissue tile *x*. The summation over patch-level class probabilities given by the CNN for all image patches belonging to a single image generates its image-level histogram; the value of its *k*-th bin is derived from Equation (2). Figure 2 represents the overview of fully supervised feature representation approach.

#### 2.3.2. Unsupervised Feature Learning

One of the main unsupervised approaches for feature representation is the bag-of-features framework. This approach was inspired by the bag-of-words scheme used for text categorization and text retrieval [27]. It consists of two main stages: In the first stage, an appropriate codebook is learned for representing the images of interest. A codebook is a visual vocabulary $V=\{c_{1},...,c_{K}\}$ including *K* representative local descriptors codified as visual words; in the second stage, each image is encoded based on each codeword’s frequencies in the image. Thus, the resulting representation of the image is a histogram of the codewords. In more detail, image classification using the bag-of-feature approach can be described in the following steps:

##### Codebook Learning

In this step, salient features of an image are identified. In the literature, different strategies have been proposed for local feature extraction from histopathology images. Popovici et al. employed the Gabor wavelets [30], and Caicedo et al. used the scale-invariant feature transform (SIFT) to extract features [27]. In this study, we employed a convolutional auto-encoder (CAE) trained in an unsupervised fashion to map each tissue tile into a low-dimensional embedding space. We then used a Gaussian mixture model (GMM) to cluster extracted features from tissue tiles into several clusters. Each cluster is considered a visual descriptor or codeblock, which are components of a codebook. Selection of the number of clusters (codebook size) is an important decision in codebook construction. This parameter should be guessed/optimized and then imported to the model as an input.

##### WSI Encoding

**Figure 3:**
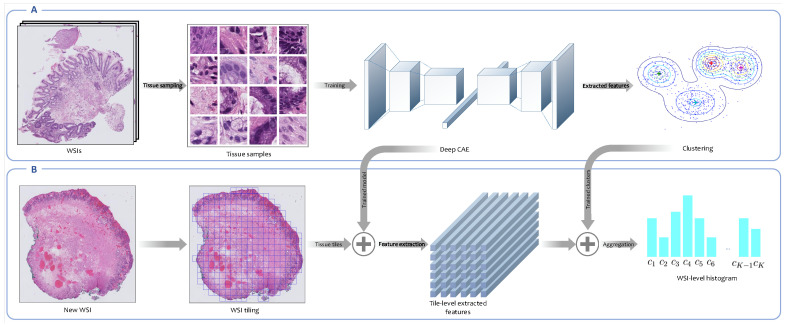
Overview of the unsupervised feature extraction framework. (**A**) This is the codebook learning phase. In this step, tissue tiles are sampled from WSIs in the training set. Neither the annotated areas nor the WSIs labels are used in this framework. An encoder is trained in an unsupervised fashion to map each high-resolution tissue tile into a low-dimensional embedding space, and then the Gaussian mixture model (GMM) is employed to cluster extracted features from tissue tiles into a number of clusters. Each cluster is indeed a morphological feature called a codeblock. The set of all codeblocks is called codebook. (**B**) This is the WSI encoding phase. In this phase, the trained convolutional autoencoder (CAE) is employed to extract embedding features from high-resolution tissue tiles derived from new WSIs. Then, the posterior probabilities of clusters constructed in the previous phase for patch-level extracted features are calculated. Finally, the posterior probabilities corresponding to all patches derived from a WSI are aggregated into a WSI-level probabilities histogram.

After codebook learning, the histogram of codeblocks’ occurrences in the set of local features of an image is considered as image representation. This concept was inspired by term frequencies (TF) in text applications [27]. The hard assignment or soft assignment of patches to the clusters can be considered, depending on which clustering algorithm is used. In the case of employing k-means (KM) clustering, which gives a hard assignment of instances to clusters, image-level histogram values are calculated based on Equation (3).

(3) $$h_{k}=\frac{1}{|X_{i}|}\sum_{x\in X_{i}}I\left(c_{p}=k\right)\;\;\;\;k=1,...,K,$$

where $h_{k}$ is the value of codeblock *k*-th in the generated histogram, and $c_{p}$ is the cluster that image patch *p*-th belongs to. If soft assignment is considered, a WSI-level histogram would be calculated according to Equation (2), where $c_{k}$ is the *k*-th cluster and $p(c_{k}|f_{\psi }\left(x\right))$ is the posterior probability of cluster *k*-th given embedding vector $f_{\psi }\left(x\right)$, which in GMM is derived from the following equation [34]:

(4) $$p\left(c_{m}|f_{\psi }\left(x\right)\right)=\frac{\pi_{m}N\left(f_{\psi }\left(x\right)|\mu_{m},\sum_{m}\right)}{\sum_{k=1}^{K}\pi_{k}N\left(f_{\psi }\left(x\right)|\mu_{k},\sum_{k}\right)},\;\;\;m=1,...,K,$$

where $\pi_{m}$ is the probability of component *m*, and N is the multivariate Gaussian distribution with mean $\mu_{m}$ and covariance matrix $\sum_{m}$. Figure 3 represents the overview of the unsupervised feature representation approach.

#### 2.3.3. Weakly Supervised Feature Learning

**Figure 4:**
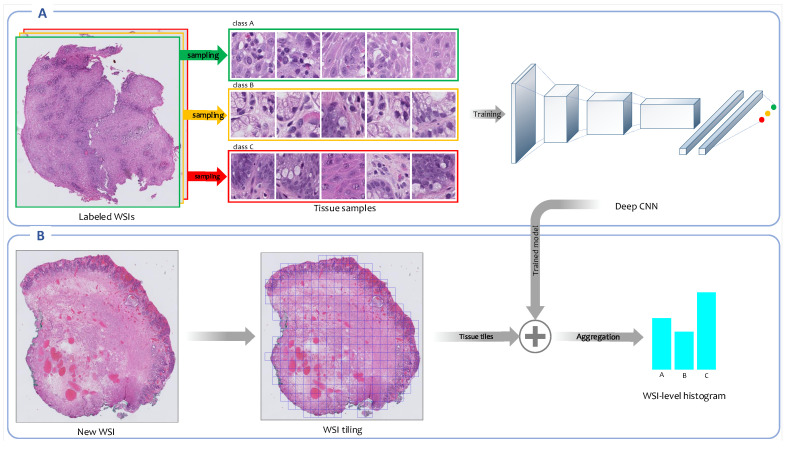
Overview of weakly supervised feature extraction framework. (**A**) A CNN is trained on high-resolution tissue tiles sampled from the labeled WSIs in the training set. This model uses only the reported diagnoses as labels for training WSIs and assumes that sampled tissue tiles have the same labels as their corresponding WSIs. (**B**) Once the training concludes, the trained model is employed to output the class’ probability distributions for each high-resolution tissue tile generated from new WSIs. The patch-level probabilities corresponding to all patches derived from a WSI are aggregated into a WSI-level probabilities histogram.

An overview of the weakly supervised feature representation approach is shown in Figure 4. In this section, multiple instance learning (MIL) [32] as a particular form of weakly supervised learning and an expectation-maximization (EM)-based method [22], which was developed as an improvement over MIL, are investigated in more detail.

##### Multiple Instance Learning

In MIL, a classifier is trained on a training set of labeled bags; each contains multiple instances. Here in the training phase, unlike the fully supervised approach in which a CNN is trained on tissue tiles sampled from pixel-wise annotated regions of WSIs, a CNN is trained on tissue tiles that are sampled from labeled WSIs. The training instances are not individually labeled, and it is assumed that each instance’s label is the same as the corresponding WSI. In this setting, some instances inside one bag might be more related to other classes of bags. These instances do not convey any relevant information about the class, providing some confusing information.

Similar to the fully supervised, in this approach, vocabulary *V* is a set of *K* classes, and mapping function M is the fully connected classifier network of CNN which outputs class probabilities $M\left(f_{\psi }\left(x\right),V\right)=\left(p\left(c_{1}|f_{\psi }\left(x\right)\right),...,p\left(c_{k}|f_{\psi }\left(x\right)\right),...,p\left(c_{K}|f_{\psi }\left(x\right)\right)\right)$ for each input tissue tile *x*. Finally, each WSI is encoded as an image-level histogram by aggregation over patch-level class probabilities given by the trained CNN for its tissue tiles.

##### Expectation-Maximization Model

In the EM method, which was proposed by [22], the main goal is to train the model over discriminative patches, i.e., those more likely to have the same labels as their corresponding WSIs. As the patch-level labels do not match the WSI labels, to avoid misleading the model by training over patches with incorrect labels, the model will be trained over patches, which are more likely to have the correct labels. It is assumed that there are hidden binary variables *z* corresponding to each tissue tile extracted from the labeled WSIs to detect discriminative patches. The hidden variable’s value is equal to 1 if and only if this tissue tile is discriminative for the corresponding WSI. In this method, at the initial E-step, it is assumed that all tissue tiles are discriminative (i.e., for all images and all tissue tiles, z=1). In the M-step, a CNN is trained over discriminative tissue tiles to maximize data likelihood. Here, vocabulary $V=\{c_{1},...,c_{K}\}$ is a set of *K* classes and the fully connected classifier network of CNN is a mapping function M which outputs class probabilities $M\left(f_{\psi }\left(x\right),V\right)=\left(p\left(c_{1}|f_{\psi }\left(x\right)\right),...,p\left(c_{k}|f_{\psi }\left(x\right)\right),...,p\left(c_{K}|f_{\psi }\left(x\right)\right)\right)$ for each input patch *x*. Then, in the E-step, the hidden variables *z* are estimated by applying Gaussian smoothing on $p(c_{k}|f_{\psi }\left(x\right))$ for all patches belong to a single image. Those patches will be considered discriminative if and only if p(z|X) is above a certain threshold. These tissue tiles are selected to continue training the CNN. Expectation and maximization steps are repeated until convergence is reached.

### 2.4. Slide-Level Inference

After encoding of WSIs using aggregation of patch-level labels, the image-level histograms are employed to train a classifier to predict the WSI-level labels. Since this decision-level fusion scheme [22] considers different patterns of the combination of patch-level labels to predict image-level class, it is more robust than the max-pooling method, which only considers the class with more tiles to predict the label of WSI. Additionally, because this image representation is generated by aggregating lots of tiles, it is very robust to misclassified tiles [22].

### 2.5. Feature Importance

Regions of interest (ROIs) detected by the models are considered to evaluate each model’s interpretability. A softmax function fit in the CNN architecture outputs each class’ probability distribution for each tissue tile in fully supervised and weakly supervised approaches. Visualizing these probabilities for every patch in a WSI generates a heatmap that highlights the attention areas associated with each class. Nevertheless, for the unsupervised approach, there is not a one-to-one correspondence between classes and codeblocks $c_{k},k=1,...,K$. In this setting, each tissue tile’s importance for class *C*, $I_{x}^{C}$ is calculated as follows:

(5) $$I_{x}^{C}=\sum_{m=1}^{K}p\left(c_{m}|f_{\psi }\left(x\right)\right)I_{c_{m}}^{C},$$

where $p(c_{m}|f_{\psi }\left(x\right))$ is the posterior probability of codeblock *m*-th given $f_{\psi }\left(x\right)$ (embedding vector of tissue tile *x*), and $I_{c_{m}}^{C}$ is the importance of the same codeblock for class *C*. We use the permutation feature importance to calculate per-class importance of each feature. In this method, each feature’s importance for a specific class is defined to be the increase in the models’ prediction error when values of that feature are randomly shuffled [35].

## 3. Experimental Evaluation

### 3.1. Patch Extraction

We employed a sliding window method on each WSI at 40× magnification to generate patches of size 128×128 pixels. Tissue tiles with less than 50% tissue sections were discarded. Image augmentation was also performed by horizontal flipping and random 90-degree rotations during training to prevent CNNs from over-fitting.

A common issue that causes bias while training the model on histopathological images is color variation. This issue, which originates from various sources, including differences in raw materials, staining protocols, and digital scanners [36], should be addressed and resolved as an essential pre-processing step before any analyses. Various solutions, such as color balancing [37], gray-scale, and stain normalization, have been proposed in the published literature to address the color variation issue. In this study, we used gray-scale images, and before converting the RGB patches to gray-scale, the stain normalization approach proposed by Vahadane et al. [36] was applied to make sure that the effect of variation of color intensity was significantly reduced.

### 3.2. Deep Models Architecture

For fully supervised and weakly supervised feature representation, we used the ResNet34 [38] architecture as our baseline architecture. We removed fully connected layers from the original network and employed the ResNet backbone as feature extractor followed by a dense layer with 1024 neurons that received the flattened output of the feature extractor. Finally, our softmax output layer was added to deliver the probability of each class. We used dropout on the fully connected layers with p = 0.5 as the regularizer.

For unsupervised feature extraction, ResNet18 was employed as a convolutional encoder. The decoder comprised convolutional and up-sampling layers to increase the size of the feature map and get back the original size of input image.

### 3.3. Experimental Setup

We used 115 WSIs from 13 patients (around 18% of the dataset) to train deep models to extract patch-level image features for WSIs encoding to associated histograms. After encoding another 535 WSIs, we employed 10-fold cross-validation, and all the models used the same groups of training and test sets for a fair evaluation. By changing some components of the approaches such as classifier type (SVM or random forest (RF)) in the decision fusion method and clustering algorithm (KM or GMM) for codebook learning in unsupervised feature learning, 10 models were evaluated in total. These models were as follows:

1. *FS-RF*: Image features were extracted using the fully supervised approach, and the random forest was employed for image-level decision fusion;
2. *FS-SVM*: Image features were extracted using the fully supervised approach, and SVM was employed for image-level decision fusion;
3. *MIL-RF*: Image features were extracted using the MIL approach, and the random forest was employed for image-level decision fusion;
4. *MIL-SVM*: Image features were extracted using the MIL approach, and SVM was employed for image-level decision fusion;
5. *EM-RF*: Image features were extracted using the EM approach, and the random forest was employed for image-level decision fusion;
6. *EM-SVM*: Image features were extracted using the EM approach, and SVM was employed for image-level decision fusion;
7. *KM-RF*: Image features were extracted using an unsupervised approach that applies the k-means clustering algorithm to learn codewords. This model employs the random forest for image-level decision fusion;
8. *KM-SVM*: Image features were extracted using an unsupervised approach that applies the k-means clustering algorithm to learn codewords. This model employs SVM for image-level decision fusion;
9. *GMM-RF*: Image features were extracted using an unsupervised approach that applies the GMM clustering algorithm to learn codewords. This model employs the random forest for image-level decision fusion;
10. *GMM-SVM*: Image features were extracted using an unsupervised approach that applies the GMM clustering algorithm to learn codewords. This model employs SVM for image-level decision fusion.

These models are summarized in Table 1.

Accuracy, the area under the ROC curve (AUC), precision, recall, and F1 score were considered as evaluation metrics for assessing the models’ performance.

### 3.4. Classification Results and Statistical Analysis

**Figure 5:**
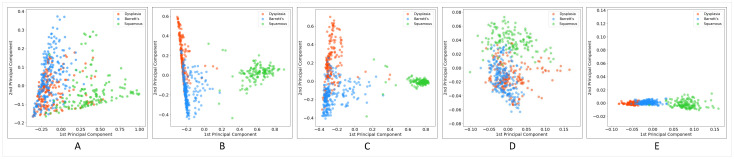
Principle component analysis (PCA) plot for WSIs encoded using (**A**) fully supervised, (**B**) multiple instance learning (MIL), (**C**) expectation-maximization (EM), (**D**) unsupervised (k-means), and (**E**) unsupervised (GMM) approaches.

The performance of a classification model was highly correlated with the degree of separability between different classes. Before applying classification algorithms on encoded WSIs, we visualized features extracted by different methods using the principal component analysis (PCA) method to understand better how well each method characterizes the histopathology images’ visual contents. Figure 5 shows the results of PCA. What can be deduced from the graphs is that in all models, squamous WSIs were encoded relatively separately from dysplastic and non-dysplastic BE. However, there is considerable confusion between these two classes. Unexpectedly, the degree of separation between images encoded by the fully supervised approach was not better than those of other methods, and the confusion between BE with dysplasia and without dysplasia and even the confusion between squamous and two other classes was higher compared to some other methods. The unsupervised approach with GMM clustering demonstrated a considerable performance in the extraction of image features, which contributed to high separability between different classes. k-means was an exception—its graph does not show acceptable separability performance, at least between two hard-to-separate classes: dysplastic and non-dysplastic BE. As the diagrams show, the MIL and EM algorithms’ performances for discriminating between classes were not much different in the domain of weakly supervised approaches. In this setting, applying the EM algorithm to detect discriminative patches to improve the training process did not lead to a significant improvement over MIL.

Classification results can further refine our findings from the PCA plots. We used 10-fold cross-validation to evaluate the models’ performances, randomly dividing 120 patients in the test set into 10-folds, using all WSIs belonging to a single patient in a fold for testing and other WSIs for training the classifier. The classification results of esophageal WSIs in three classes of squamous, dysplastic BE, and non-dysplastic BE for different models are summarized in Table 2. The reported values are averages with 95% confidence intervals. For computing confidence intervals, numbers greater than one were truncated to 1.

As the number of clusters is a key parameter in the performance of clustering methods, we evaluated different numbers of clusters (codeblocks) for both k-means and GMM to pick a decent number of codeblocks given our dataset and type of classifier. We used weighted AUC as an evaluation metric. Table 3 summarizes the results of the evaluation of different numbers of clusters for k-means and GMM. These results demonstrated that the optimal feature number was as follows: 200 features for the combination of k-means and SVM, 100 features for the combination of k-means and random forest, and 150 features for GMM.

**Figure 6:**
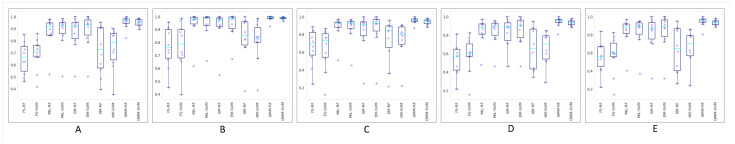
Boxplots of the 10-fold cross-validation results for weighted (**A**) accuracy, (**B**) area under the ROC curve (AUC), (**C**) precision, (**D**) recall, and (**E**) F1 score in different models.

Figure 6 shows boxplots of the 10-fold cross-validation for different metrics. As shown, GMM-RF and GMM-SVM performed better than the other models, and as PCA plots show and boxplots confirm, the performances of classification models on WSIs encoded by the fully supervised approach and also WSIs encoded by employing k-means clustering were poorer than those of the other models.

In terms of the classification model, results show that there is not a significant difference between the performances of SVM and random forest given the same set of encoded WSIs. In other words, choosing the type of classifier is not a critical decision as opposed to deciding on the feature extraction approach.

### 3.5. Visualization of ROIs on WSIs

**Figure 7:**
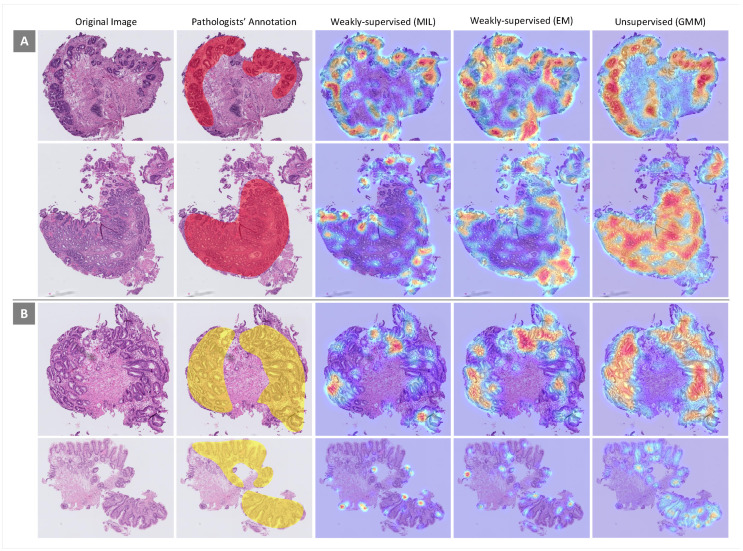
Heatmaps generated by different feature extraction approaches for some samples from (**A**) dysplastic Barrett’s esophagus (BE) and (**B**) non-dysplastic BE. Area of attention is shown in red.

For more scrutiny about the models’ performances, heatmaps using different feature extraction approaches on WSIs with dysplastic BE and non-dysplastic BE were considered. In this evaluation, the performances of weakly supervised (MIL and EM) and unsupervised (GMM) approaches, which have better performances in the classification of WSIs, were examined. Some heatmaps for WSIs from both classes generated by the three approaches mentioned above are presented in Figure 7. In each heatmap, image regions that are more important given the associated classes are in red. As can be seen, the performance of the unsupervised approach in ROI detection is far better than the performances of the weakly supervised approaches.

## 4. Discussion

In this comparative study, the performances of different deep learning-based approaches for extracting image features from tissue tiles for identification of dysplastic and non-dysplastic BE on whole-slide tissue histopathology images were evaluated. The results demonstrated the capability of the unsupervised approach in extracting relevant image features from WSIs if an appropriate setting is chosen. This is important because the unsupervised approach without utilizing any manually annotated image or even image-level diagnosis learned a discriminative representation of esophageal WSIs.

By contrast, the fully supervised feature extraction approach did not achieve as high a performance as the other two approaches. Although the fully supervised approach used the same deep learning model as the weakly supervised approach, difficulties in obtaining accurate annotations on WSIs may explain the relatively poor performance of the fully supervised approach. Specifically, distinguishing non-dysplastic BE from low-grade dysplasia can be challenging even for human pathologists, and annotations involving confusing areas may muddle the inputs for the fully supervised model.

In the unsupervised approach, a CAE was applied to map tissue tiles to an embedding space to reduce the dimensionality of raw image features. Then, the embedding vectors were clustered into using a clustering algorithm independently of the first step. Although employing GMM for the soft assignment of embedded features to clusters and encoding WSIs generated promising results, the assumptions underlying the dimensionality reduction techniques are generally independent of the clustering techniques’ assumptions. Thus, there is no theoretical guarantee that the network would learn feasible representations. End-to-end training of such a model can be an improvement over the unsupervised approach applied in this study. Applying such a model ensures that features are learned that lead to the best results in the WSI classification phase.

A deep learning-based model for detecting and locating dysplastic and non-dysplastic BE patterns on histopathologic images has a wide variety of applications in clinical settings. Such a model can be integrated into clinical information management systems as a decision support system. These support systems can provide clinicians with “possible” diagnoses or improve confidence in their assessments via providing second opinions for prognostic decision-making of more challenging histopathological patterns. Successful implementation of this system can support a more accurate classification of pre-malignant diseases of the esophagus.

This study has some limitations. First, all biopsy images used for this study were collected from a single center and scanned with the same equipment. Thus, such data might not be representative of the entire range of histopathological patterns in patients worldwide. Collaboration with other medical centers and collecting more images would allow us to refine our model using a more diverse dataset. Furthermore, in this study, we applied the gray-scale images to reduce color variation to prevent the model from being misled. Repeating this study with color images while at the same time applying an approach to mitigate the color variation problem could be another potential area of future work.

## 5. Conclusions

In this paper, we performed a comparative study on different feature representation approaches, including unsupervised, weakly supervised, and fully supervised approaches to classify precursors to esophageal cancer using whole-slide histopathology images. We used a two-step process, in which, in the first step, a feature representation of WSIs was learned as an image-level histogram, and a decision fusion model was trained on histograms in the second step to output the final labels of new WSIs. Considering different feature extraction approaches in the first step and two different classification models (i.e., SVM and random forest) in the second step, 10 models were evaluated on an independent test set of 535 WSIs from 120 patients. The results showed that applying the unsupervised approach can lead to extracting more relevant image features from WSIs, and consequently, better identifying dysplastic and non-dysplastic BE. Employing a CAE for mapping tissue tiles in an embedding space while aiming for dimensionality reduction, and then their soft assignment to a relatively large number of clusters using GMM can generate a discriminative representation of esophageal WSIs. Applying a classification algorithm in such representations accurately predicted the labels of WSIs. Highlighting the histopathological patterns identified by the models that contributed to the WSI classification and comparing them with the same images annotated by our pathologist annotators demonstrated a comparable diagnostic performance of the unsupervised feature representation approach. Nevertheless, despite having relatively good classification results, weakly supervised approaches showed a poor performance in locating regions of interest on WSIs. Thus, the model trained on histopathological features learned by the unsupervised approach, if confirmed in clinical trials, could be employed to improve diagnostic procedures of precursors to esophageal cancer.

## Figures and Tables

**Table 1:** Summary of models.

Feature Extraction Approach	Clustering Algorithm	Classification Algorithm	Model
fully supervised	-	Random Forest	FS-RF
fully supervised	-	SVM	FS-SVM
weakly supervised (MIL)	-	Random Forest	MIL-RF
weakly supervised (MIL)	-	SVM	MIL-SVM
weakly supervised (EM)	-	Random Forest	EM-RF
weakly supervised (EM)	-	SVM	EM-SVM
unsupervised	k-means	Random Forest	KM-RF
unsupervised	k-means	SVM	KM-SVM
unsupervised	GMM	Random Forest	GMM-RF
unsupervised	GMM	SVM	GMM-SVM

**Table 2:** Results of WSI classification.

Models	Classes	Metrices				
		Accuracy	AUC	Precision	Recall	F1 score
FS-RF	Dysplastic BE	0.563[0.454,0.671]	0.616[0.489,0.743]	0.395[0.211,0.578]	0.451[0.319,0.584]	0.367[0.261,0.472]
	Non-dysplastic BE	0.655[0.563,0.746]	0.758[0.653,0.863]	0.565[0.360,0.770]	0.555[0.384,0.726]	0.520[0.360,0.680]
	Squamous	0.857[0.755,0.958]	0.934[0.866,1.000]	0.760[0.565,0.954]	0.732[0.566,0.898]	0.720[0.558,0.881]
	Weighted Average	0.655[0.560,0.751]	0.761[0.647,0.876]	0.655[0.507,0.802]	0.537[0.416,0.657]	0.554[0.430,0.678]
FS-SVM	Dysplastic BE	0.619[0.480,0.758]	0.661[0.481,0.840]	0.434[0.246,0.622]	0.565[0.379,0.751]	0.455[0.303,0.607]
	Non-dysplastic BE	0.663[0.551,0.775]	0.778[0.665,0.892]	0.542[0.323,0.762]	0.418[0.162,0.673]	0.424[0.217,0.632]
	Squamous	0.888[0.811,0.964]	0.921[0.842,1.000]	0.741[0.565,0.916]	0.860[0.710,1.000]	0.779[0.634,0.923]
	Weighted Average	0.689[0.593,0.784]	0.773[0.644,0.902]	0.637[0.466,0.807]	0.585[0.448,0.721]	0.572[0.423,0.721]
MIL-RF	Dysplastic BE	0.842[0.739,0.945]	0.924[0.835,1.000]	0.756[0.584,0.929]	0.761[0.526,0.996]	0.694[0.524,0.863]
	Non-dysplastic BE	0.844[0.742,0.945]	0.926[0.835,1.000]	0.816[0.657,0.976]	0.820[0.710,0.930]	0.793[0.692,0.893]
	Squamous	0.990[0.978,1.000]	1.000[0.999,1.000]	0.964[0.899,1.000]	0.984[0.955,1.000]	0.971[0.933,1.000]
	Weighted Average	0.874[0.778,0.971]	0.939[0.856,1.000]	0.877[0.781,0.974]	0.838[0.738,0.938]	0.831[0.716,0.947]
MIL-SVM	Dysplastic BE	0.845[0.736,0.954]	0.918[0.828,1.000]	0.744[0.592,0.895]	0.775[0.535,1.000]	0.707[0.534,0.880]
	Non-dysplastic BE	0.847[0.738,0.956]	0.935[0.858,1.000]	0.840[0.689,0.991]	0.808[0.696,0.920]	0.799[0.694,0.905]
	Squamous	0.989[0.978,1.000]	0.998[0.995,1.000]	0.983[0.946,1.000]	0.968[0.931,1.000]	0.974[0.949,0.999]
	Weighted Average	0.876[0.774,0.978]	0.938[0.862,1.000]	0.868[0.758,0.977]	0.841[0.733,0.948]	0.831[0.704,0.957]
EM-RF	Dysplastic BE	0.837[0.719,0.954]	0.896[0.783,1.000]	0.668[0.436,0.901]	0.747[0.480,1.000]	0.676[0.439,0.912]
	Non-dysplastic BE	0.836[0.717,0.954]	0.915[0.818,1.000]	0.809[0.649,0.969]	0.817[0.676,0.958]	0.794[0.661,0.928]
	Squamous	0.985[0.974,0.996]	0.998[0.994,1.000]	0.958[0.893,1.000]	0.963[0.925,1.000]	0.957[0.918,0.996]
	Weighted Average	0.865[0.760,0.971]	0.923[0.825,1.000]	0.828[0.670,0.986]	0.829[0.713,0.944]	0.814[0.671,0.958]
EM-SVM	Dysplastic BE	0.858[0.738,0.979]	0.900[0.805,0.994]	0.757[0.524,0.990]	0.757[0.486,1.000]	0.709[0.474,0.945]
	Non-dysplastic BE	0.859[0.739,0.980]	0.938[0.870,1.000]	0.846[0.688,1.000]	0.863[0.733,0.994]	0.834[0.705,0.963]
	Squamous	0.983[0.970,0.996]	1.000[1.000,1.000]	0.958[0.894,1.000]	0.964[0.922,1.000]	0.957[0.920,0.995]
	Weighted Average	0.883[0.775,0.991]	0.935[0.861,1.000]	0.852[0.691,1.000]	0.850[0.732,0.969]	0.834[0.687,0.981]
KM-RF	Dysplastic BE	0.660[0.488,0.831]	0.778[0.651,0.905]	0.517[0.238,0.796]	0.516[0.232,0.801]	0.449[0.212,0.686]
	Non-dysplastic BE	0.682[0.522,0.843]	0.793[0.642,0.943]	0.665[0.440,0.890]	0.727[0.539,0.915]	0.626[0.440,0.812]
	Squamous	0.954[0.910,0.998]	0.996[0.990,1.000]	0.877[0.725,1.000]	0.976[0.946,1.000]	0.907[0.806,1.000]
	Weighted Average	0.720[0.575,0.865]	0.836[0.715,0.957]	0.728[0.547,0.908]	0.648[0.484,0.812]	0.631[0.453,0.810]
KM-SVM	Dysplastic BE	0.676[0.542,0.809]	0.754[0.642,0.867]	0.512[0.322,0.702]	0.632[0.412,0.852]	0.509[0.353,0.662]
	Non-dysplastic BE	0.705[0.565,0.845]	0.776[0.641,0.912]	0.672[0.468,0.876]	0.643[0.499,0.788]	0.615[0.461,0.769]
	Squamous	0.939[0.884,0.995]	0.976[0.935,1.000]	0.852[0.695,1.000]	0.946[0.883,1.000]	0.880[0.768,0.991]
	Weighted Average	0.733[0.608,0.857]	0.812[0.695,0.929]	0.743[0.597,0.889]	0.660[0.531,0.790]	0.664[0.527,0.802]
GMM-RF	Dysplastic BE	0.948[0.907,0.989]	0.985[0.967,1.000]	0.921[0.834,1.000]	0.929[0.843,1.000]	0.914[0.856,0.972]
	Non-dysplastic BE	0.941[0.903,0.979]	0.983[0.965,1.000]	0.892[0.776,1.000]	0.947[0.912,0.982]	0.910[0.840,0.981]
	Squamous	0.993[0.984,1.000]	0.999[0.997,1.000]	0.985[0.960,1.000]	0.988[0.959,1.000]	0.986[0.967,1.000]
	Weighted Average	0.952[0.915,0.989]	0.986[0.970,1.000]	0.955[0.930,0.980]	0.941[0.903,0.979]	0.942[0.904,0.981]
GMM-SVM	Dysplastic BE	0.937[0.913,0.961]	0.988[0.980,0.997]	0.814[0.708,0.921]	0.976[0.948,1.000]	0.879[0.811,0.946]
	Non-dysplastic BE	0.931[0.909,0.954]	0.973[0.959,0.987]	0.937[0.882,0.991]	0.862[0.814,0.910]	0.895[0.858,0.933]
	Squamous	0.994[0.987,1.000]	1.000[1.000,1.000]	1.000[1.000,1.000]	0.959[0.908,1.000]	0.978[0.950,1.000]
	Weighted Average	0.950[0.928,0.972]	0.986[0.977,0.995]	0.942[0.921,0.964]	0.931[0.909,0.954]	0.933[0.912,0.954]

**Table 3:** Area under the ROC curve (AUC) comparison among different numbers of codeblocks.

Number of Codeblock	KM		GMM	
	RF	SVM	RF	SVM
3	0.583[0.454,0.713]	0.594[0.440,0.748]	0.551[0.465,0.637]	0.651[0.538,0.764]
5	0.690[0.578,0.802]	0.698[0.578,0.817]	0.681[0.586,0.775]	0.698[0.597,0.799]
10	0.799[0.685,0.913]	0.795[0.682,0.907]	0.812[0.716,0.908]	0.789[0.685,0.894]
20	0.806[0.676,0.936]	0.801[0.682,0.920]	0.894[0.815,0.973]	0.827[0.747,0.906]
50	0.821[0.690,0.952]	0.793[0.664,0.921]	0.931[0.875,0.987]	0.843[0.764,0.921]
100	0.836[0.715,0.957]	0.796[0.683,0.909]	0.956[0.914,0.997]	0.922[0.872,0.972]
150	0.824[0.968,0.949]	0.809[0.685,0.933]	0.986[0.970,1.000]	0.986[0.977,0.995]
200	0.833[0.711,0.957]	0.812[0.695,0.929]	0.984[0.967,1.000]	0.978[0.962,0.994]
300	0.830[0.701,0.959]	0.812[0.693,0.931]	0.984[0.968,1.000]	0.983[0.971,0.995]

## Abbreviations

The following abbreviations are used in this manuscript:

BE	Barrett’s Esophagus
WSI	Whole-Slide Images
H&E	Hematoxylin and Eosin
HD-WLE	High-Definition White-Light Endoscopy
NBI	Narrow Band Imaging
CNN	Convolutional Neural Networks
SIFT	Scale-Invariant Feature Transform
CAE	Convolutional Auto-Encoder
GMM	Gaussian Mixture Model
TF	Term Frequencies
MIL	Multiple Instance Learning
EM	Expectation-Maximization
ROI	Regions of Interest
FS	Fully Supervised
RF	Random Forest
KM	k-Means Clustering
AUC	Area Under the ROC Curve
PCA	Principle Components Analysis
